# Letalität und Risikofaktoren für einen schweren Verlauf der COVID-19-Pneumonie

**DOI:** 10.1007/s10405-020-00349-y

**Published:** 2020-10-26

**Authors:** Holger Flick

**Affiliations:** Universitätsklinik für Innere Medizin, Klinische Abteilung für Pulmonologie, LKH-Univ. Klinikum Graz, Augenbruggerplatz 15, 8036 Graz, Österreich

**Keywords:** SARS-CoV‑2, Krankenhaus, Mortalität, Risikoeinschätzung, Biomarker, SARS-CoV‑2, Hospital, Mortality, Risk estimation, Biomarkers

## Abstract

Die Schwere einer SARS-CoV-2-Pneumonie, ihr Verlauf, die Letalität und Mortalität werden multifaktoriell durch den unmittelbaren Parenchymschaden im Bereich der Lungen (inklusive der Pulmonalgefäße), vorbestehende Komorbiditäten, extrapulmonale Komplikationen, sekundäre Infektionen und die Qualität der verfügbaren medizinischen Versorgung bedingt. Diesbezüglich ist COVID-19 vergleichbar mit anderen schweren ambulant erworbenen Pneumonien durch konventionelle Erreger, auch wenn sich die Pathogenese unterscheidet. Die Letalität von hospitalisierten COVID-19-Patienten beträgt näherungsweise 20 % (damit höher als bei anderen Pneumonieerregern), bei intensivpflichtigen Patienten 30–40 % und von invasiv beatmeten Patienten ca. 50 %. Für die Letalität ausschlaggebende Risikofaktoren sind hohes Alter, Übergewicht, männliches Geschlecht und typische altersabhängige kardiopulmonale Grunderkrankungen. Die klinische Risikoeinschätzung im Krankenhaus sollte im Wesentlichen entsprechend den gültigen Pneumonieleitlinien erfolgen. Die Wertigkeit von COVID-19-spezifischen laborchemischen Surrogatmarkern zur Risikoeinschätzung und Behandlungsoptimierung ist noch nicht ausreichend beurteilbar.

## Abstract (en)

The severity of severe acute respiratory syndrome coronavirus 2 (SARS-CoV-2) pneumonia, the course, fatality and mortality are multifactorial and attributable to the immediate parenchymal damage in the region of the lungs (including pulmonary vessels), pre-existing comorbidities, extrapulmonary complications, secondary infections and the quality of the available medical care. In this respect, coronavirus disease 2019 (COVID-19) is comparable with other severe community-acquired forms of pneumonia caused by conventional pathogens, even if the pathogenesis is different. The fatality of hospitalized COVID-19 patients is approximately 20% (and therefore higher than for other pneumonia pathogens), in intensive care patients 30–40% and in invasively ventilated patients ca. 50%. Risk factors that are decisive for the fatality are old age, overweight, male gender and typical age-related cardiopulmonary underlying diseases. The clinical risk estimation in hospital should essentially be carried out in accordance with the valid guidelines on pneumonia. The value of laboratory surrogate markers specific for COVID-19 for risk estimation and treatment optimization cannot yet be adequately assessed.

Die COVID-19-Pandemie breitete sich Anfang 2020 rasant aus und erfasste innerhalb kürzester Zeit alle Weltregionen. Die hohe Zahl an Infizierten und die hohe Letalitätsrate haben dazu geführt, dass nach 9 Monaten weltweit fast 1.000.000 Menschen an einer SARS-CoV-2-Infektion gestorben sind. Andererseits wurde in dieser Zeit durch wissenschaftliche Beobachtungen und Studien eine beeindruckende Menge an Informationen über SARS-CoV‑2 und die damit assoziierte Erkrankung („coronavirus disease 2019“ [COVID-19]) gesammelt und ausgewertet. Aufgrund dieser Daten kann inzwischen beurteilt werden, welche COVID-19-Patienten durch die Infektion vital gefährdet sind und von Präventivmaßnahmen besonders profitieren können.

## Begriffsbestimmung schwere COVID-19-Pneumonie, Mortalität und Letalität

Mortalität und Letalität beschreiben Sterblichkeitsraten, aber entweder mit Bezug auf eine Gesamtpopulation oder bezogen auf die Gesamtheit der von einer Erkrankung betroffenen Patienten. Die Begriffe sollten nicht synonym verwendet werden. Das Robert Koch-Institut definiert Mortalität und Letalität wie folgt [19]:

### Mortalität (engl.: „mortality“).

Sterblichkeit in einer Population in einem bestimmten Zeitraum. Krankheitsspezifische Mortalität (engl.: „disease specific mortality“): Verhältnis aus Anzahl der in einer Population in einem Zeitraum an einer bestimmten Krankheit Gestorbenen zur Anzahl der Personen, die dieser Population angehören (z. B. pro 100.000 Einwohner).

### Letalität (engl.: „fatality“).

Beschreibt das krankheitsbezogene Sterberisiko für Erkrankte unter definierten Bedingungen. Die fallbezogene Fatalitätsrate (engl.: „case fatality rate“ [CFR]) stellt die Zahl der Fälle einer bestimmten Krankheit mit tödlichem Verlauf in einem Zeitraum ins Verhältnis zur Zahl der insgesamt an dieser Krankheit Erkrankten im gleichen Zeitraum. Die CFR wird üblicherweise als Prozentsatz ausgewiesen.

In klinischen Studien und Publikationen werden diese Begrifflichkeiten häufig unscharf verwendet. Bei Studien mit Bezug zur COVID-19-Sterblichkeit wird inhaltlich in der Regel die Letalität und nicht die Mortalität beschrieben.

### Schweregrad einer COVID-19-Infektion.

Das Letalitätsrisiko jeder ambulant erworbenen Pneumonie (CAP) ist unabhängig vom Erreger multifaktoriell durch die unmittelbare alveoläre, interstitielle und vaskuläre Schädigung der Lunge, sekundäre Infektionen, extrapulmonale Komplikationen, Alter und noch wichtiger von vorbestehenden Komorbiditäten sowie der Qualität der medizinischen Versorgung abhängig [3, 4]. Diese allgemeinen Rahmenbedingungen sind auch für den neuen CAP-Erreger SARS-CoV‑2 zutreffend und primär zu berücksichtigen.

Der Schweregrad einer CAP hat therapeutische Konsequenzen und sollte frühzeitig eingeschätzt und im Verlauf reevaluiert werden. Zur Orientierung dienen die aktuellen CAP-Leitlinien, die aus oben genannten Gründen während der COVID-19-Pandemie und auch für die SARS-CoV-2-CAP von großer Relevanz sind (z. B. S3-Leitlinie, letztes Update 2016) [20, 21].

Der Schweregrad einer CAP hat therapeutische Konsequenzen

Bei einer leichtgradigen CAP liegt ein niedriges Letalitätsrisiko vor, und eine ambulante Therapie ist gerechtfertigt. Eine mittelschwere CAP wird unter regulären Versorgungsbedingungen dagegen im Krankenhaus und die sehr schwere CAP auf Intensiv- oder Intermediate-care-Stationen (ITS, IMC) behandelt.

Die multimodale Schweregradbestimmung (Letalitätsrisikoabschätzung) einer CAP berücksichtigt eine Vielzahl von klinischen Parametern, die mittels evaluierter Pneumoniescores (beispielsweise Kombination aus CRB-65, der Bestimmung der peripheren Sauerstoffsättigung [S_p_O_2_] oder des Oxygenierungsindex [F_i_O_2_/P_a_O_2_] und den IDSA[Infectious Diseases Society of America]/ATS[American Thoracic Society]-Kriterien) bei der Erstuntersuchung systematisch erfasst und im Verlauf reevaluiert werden müssen. Zusätzlich werden relevante Komorbiditäten (und deren akute Dekompensation), aber auch die bisherige Funktionalität des Patienten berücksichtigt [22, 23].

Dieses multimodale Konzept der Risikoabschätzung wird mit wenigen Anpassungen auch zur Evaluierung von COVID-19-Infektionen empfohlen [21].

In COVID-19-Epizentren (beispielsweise Lombardei oder Wuhan) wurde aufgrund der passageren katastrophenmedizinischen Situation die CAP-Schweregradbestimmung teilweise auf nur wenige klinische Parameter reduziert (z. B. Dyspnoe, S_p_O_2_ oder pO_2_, Atemfrequenz und Ausmaß der Infiltrate im Röntgen) [24]. Eine solche Reduktion war in Deutschland oder Österreich zu keinem Zeitpunkt der Pandemie notwendig oder gerechtfertigt. Auch die isolierte Verwendung modifizierter CRB-65-Scores ohne Erfassung des O_2_[Sauerstoff]-Bedarfs bzw. der aktuellen pulmonalen Oxygenierungsfunktion unterschätzt die Letalitätswahrscheinlichkeit und ist in unserer Versorgungsrealität nicht empfehlenswert [22, 23].

## Krankenhausletalität bei COVID-19 im Vergleich zu anderen ambulant erworbenen Pneumonien in Deutschland und Österreich

Viele SARS-CoV-2-Infektionen verlaufen relativ mild. Zu Beginn der Pandemie mussten aber laut dem European Centre for Disease Prevention and Control (ECDC) in Europa (EU/EEA und UK) 28 % aller bestätigten COVID-19-Fälle wegen schweren Verlaufs hospitalisiert werden, und im Krankenhaus benötigten 14 % aller COVID-19-Patienten eine intensivmedizinische Behandlung (Stand August 2020). Darüber hinaus sind in Europa schätzungsweise 24 % aller hospitalisierten COVID-19-Patienten verstorben („country range“: 0,5–38,0 %) [25].

Im Rahmen der seit August 2020 in Europa wieder zunehmenden Infektionszahlen (sog. zweite Welle) werden aktuell (Stand Ende September 2020) weniger schwere Verläufe registriert als zu Beginn der Pandemie. So sind in Österreich derzeit 8500 Menschen als mit SARS-CoV‑2 infiziert gemeldet, jedoch davon nur 362 Patienten (4 %) hospitalisiert. Die Gründe hierfür sind vielschichtig (beispielweise viele junge Sommerurlaubreiserückkehrer aus COVID-19-Risikogebieten, erhöhte Testkapazitäten, Risikogruppen besser geschützt als zu Beginn der Pandemie, Risikoverhalten v. a. bei jüngeren Erwachsenen ausgeprägt) und unterliegen einer nur teilweise vorhersehbaren Dynamik. Die Rate intensivpflichtiger Patienten ist jedoch unverändert relativ hoch (aktuell in Österreich 23 % aller hospitalisierten Patienten), was die Gefährlichkeit von COVID-19 unverändert untermauert [26].

Im Gegensatz zu anderen europäischen Ländern konnten in Deutschland und Österreich schwer erkrankte COVID-19-Patienten durchgängig und flächendeckend entsprechend den üblichen medizinischen Standards versorgt werden. In beiden Ländern waren ausreichend Krankenhauskapazitäten inklusive geschultes Personal und Intensiv‑/Beatmungsbetten verfügbar. Unter diesen optimalen Versorgungsbedingungen wurden in Deutschland die klinischen Daten von fast 12.000 hospitalisierten Patienten gesammelt und in 2 Studien veröffentlicht [1, 2]. Die deutschen COVID-19-Daten können nun mit deutschen und österreichischen CAP-Studiendaten aus den vorangegangenen Jahren verglichen werden (Tab. 1). Hierbei wird deutlich, dass die allgemeine Krankenhausletalität bei COVID-19 höher ausfällt als bei anderen CAP-Formen (16,7–22,2 % vs. 3,2–18,5 %), was die vitale Bedrohung durch COVID-19 unterstreicht. Die ICU(„intensive care unit“)-Letalität von COVID-19-Patienten scheint sich jedoch nicht wesentlich von anderen Pneumonieerregern abzuheben.

**Table 1:** 

CAP	Jahr^a^	*n*	Hospitalletalität (%)	ICU-Letalität (%) (Gesamt)	ICU-Letalität (Non-IMV)	ICU-Letalität (NIV)	ICU-Letalität (IMV)
COVID-19 [1]^d^	2020	1904	16,7	29,1	23,0 %	–	32,8 %
COVID-19 [2]^d^	2020	10.021	22,2	–	–	44,8 %	52,8 %
CAP allgemein [3]^d^	2012	442.831	9,5	–	–	–	–
CAP allgemein [4]^d^	2009	388.406	14,1	–	–	–	–
CAP allgemein [5]^d^	2015	3427	3,2^b^	26,6^b,c^	–	–	–
*L. pneumonia* [6]^d^	2008	94	18,5	–	–	–	–
Virale CAP [7]^d^	2019	61	14,8	–	–	–	–
Influenza A/B [8]^e^	2020	95	12,6	–	–	–	–
Influenza H1N1 [9]^e^	2011	540	–	26,5	–	–	–
Influenza A/B [10]^d^	2019	51	–	41,2	–	–	–

*COVID-19* „coronavirus disease 2019“, *NIV* „non-invasive ventilation“, *IMV* „invasive ventilation“, *L. pneumonia Legionella pneumonia*

^a^Publikationsjahr

^b^30-Tage-Mortalität

^c^ICU-Patienten mit NIV, IMV oder vasopressorischer Unterstützung innerhalb der ersten 7 Tage der Krankenhausbehandlung

^d^Deutschland

^e^Österreich

## Risikofaktoren für einen schweren Verlauf einer COVID-19-Pneumonie

Die deutschen COVID-19-Daten belegen, dass die Schwere und das Letalitätsrisiko auch bei COVID-19-Patienten vom Alter, von dem Geschlecht, der Zahl und Schwere der Begleiterkrankungen abhängen und sich bezüglich ihrer prädisponierenden Faktoren nicht wesentlich von anderen deutschen CAP-Patienten unterscheiden (Tab. 2 und 3). Die stärksten Risikofaktoren für einen tödlichen COVID-19-Verlauf sind in Deutschland hohes Alter (Hazard Ratio [HR] 4,11; 95 %-KI [Konfidenzintervall] 2,57–6,58; verglichen >79 Jahre mit <60 Jahre), vorbekannte Lungenerkrankung (HR 1,61; 95 %-KI 1,20–2,16) und männliches Geschlecht (HR 1,45; 95 %-KI 1,15–1,83) [1]. Diese Letalitätsrisikofaktoren finden sich auch in größeren Metaanalysen von internationalen COVID-19-Studien wieder, in denen aber zusätzlich kardiovaskuläre Erkrankungen, Übergewicht, maligne Erkrankungen, Diabetes und teilweise das Rauchen als wesentlich beschrieben werden (Tab. 4).

**Table 2:** 

	**COVID-19 **[1, 2]	**CAP andere Erreger **[4]
*Alter in Jahren (Median)*	72–73	76
*Letalität entsprechend dem Alter*		
<60 Jahre	2–6 %	1–7 %
60 bis 69 Jahre	9–15 %	10 %
70 bis 79 Jahre	22–27 %	14 %
>79 Jahre	30–38 %	19–25 %
*Komorbiditäten*		
Herzerkrankungen	20–36 %	19 %
Lungenerkrankungen	12–14 %	21 %
Diabetes mellitus	15–30 %	12 %
Nierenerkrankungen	23 %	9 %
Malignom	5 %	5 %

**Table 3:** 

	**COVID-19** [11, 12]	**CAP (andere Erreger)** [13, 14]
*Alter in Jahren (Median)*	82	62
*Komorbiditäten*		
Arterieller Hypertonus	40–66 %	57 %
Diabetes	20–30 %	31 %
Kardiovaskuläre Erkrankungen (allgemein)	23 %	20–38 %
Neurologische Erkrankungen	13 %	11–16 %
Karzinome	2–17 %	28 %
Chronische Niereninsuffizienz	21 %	3–27 %
Chronische Lungenerkrankungen	8–17 %	22–24 %
Demenz	20 %	%

**Table 4:** 

	**Odds Ratio (95** **%-KI)**
*Geschlecht männlich [* 15*]*	1,2 (1,07–1,23)
*Alter >50 Jahre [* 17*]*	8,7 (5,10–14,9)
*BMI >25 [* 18*]*	3,7 (1,54–8,83)
*Komorbiditäten*	
Herzinsuffizienz [17]	27,8 (6,3–122,9)
Raucher [17]	13,6 (2,9–63,5)
Lungenerkrankungen [17]	3,4 (1,4–8,1)
Kardiovaskuläre Erkrankungen (allgemein) [17]	2,7 (1,6–4,8)
Hypertonus [17]	2,6 (1,9–3,7)
Malignom [16]	1,9 (1,17–3,15)
Diabetes [17]	1,7 (1,0–2,8)
Zerebrovaskuläre Erkrankungen [15]	1,4 (1,14–1,77)

*BMI* Body-Mass-Index, *CI* Konfidenzintervall

Zu beachten ist, dass die Letalität der COVID-19-Pneumonie bei älteren Patienten (>70 Jahre) die zu erwartende altersspezifische Letalität von anderen Pneumonieerregern deutlich übersteigt (Tab. 2) und ältere Patienten mit COVID-19-Pneumonie somit als überdurchschnittlich vulnerabel zu betrachten sind. Dabei scheint nicht nur das Alter, sondern auch das Vorliegen der oben genannten altersabhängigen Erkrankungen relevant zu sein [27].

Ältere Patienten mit COVID-19-Pneumonie sind als überdurchschnittlich vulnerabel zu betrachten

In einer kürzlich publizierten Studie aus den USA wurden die letal verlaufenden COVID-19-Pneumonien bei 18 bis 34 Jahre alten Patienten analysiert. Es zeigte sich, dass der entscheidende Risikofaktor (Odds Ratio [OR] 2,30; 95 %-KI, 1,77–2,98; *p* < 0,001) für einen letalen Verlauf oder eine mechanische Ventilation eine „morbid obesity“ war, die bei 41 % der Beatmeten bzw. Verstorbenen vorlag. Andere mit dem Übergewicht assoziierte Risikofaktoren verloren neben dem BMI (Body-Mass-Index) ihre statistische Unabhängigkeit (z. B. Diabetes mellitus). Lediglich die arterielle Hypertonie und das männliche Geschlecht blieben als unabhängiger Risikofaktor ebenfalls bestehen [28]. Dieses Risikoprofil spiegelt sich auch bei den älteren Patienten mit COVID-19-Pneumonie wider, wird dort jedoch durch die mittlerweile eingetretenen chronischen Krankheiten und ihre Sekundärkomplikationen überlagert.

## Biomarker für einen schweren Verlauf der COVID-19-Pneumonie

In einer Vielzahl von Studien und Metaanalysen wurden laborchemische prädiktive Marker identifiziert, die mit einer erhöhten COVID-19-Letalität assoziiert sind (unter anderem CRP [C-reaktives Protein], IL[Interleukin]-6, D‑Dimer, LDH [Laktatdehydrogenase], CK [Kreatinkinase], Troponin) [17, 29]. Die Frage nach sinnvollen COVID-19-spezifischen Grenzwerten („cut off“) und nach der klinischen Relevanz dieser Biomarker für die Risikoevaluierung und therapeutische Entscheidungen in den verschiedenen Altersgruppen ist noch nicht ausreichend beantwortet.

## Fazit für die Praxis

- Von schweren Verläufen sind v. a. komorbide ältere Menschen betroffen.
- Zusätzliche Risikofaktoren sind Übergewicht, männliches Geschlecht, chronische Herz- und Lungenkrankheiten und maligne Grunderkrankungen.
- Hospitalisiert werden überwiegend ältere Patienten (Altersmedian ca. 70 Jahre).
- Die Krankenhausletalität liegt im Durchschnitt bei 20 % und somit höher als bei anderen ambulant erworbenen Pneumonien.
- Die Sterblichkeit auf den Intensivstationen variiert und liegt näherungsweise bei 30 % und ist somit ähnlich hoch wie bei schweren Pneumonien durch andere ambulant erworbene Erreger.
- Invasiv beatmete Patienten sterben in etwa der Hälfte der Fälle. Auch hier ist die Letalität altersabhängig.
- Der Stellenwert von laborchemischen Biomarkern ist derzeit noch nicht ausreichend definiert.
